# Genetic structure of urban and non-urban populations differs between two common parid species

**DOI:** 10.1038/s41598-021-89847-4

**Published:** 2021-05-17

**Authors:** Marcin Markowski, Piotr Minias, Mirosława Bańbura, Michał Glądalski, Adam Kaliński, Joanna Skwarska, Jarosław Wawrzyniak, Piotr Zieliński, Jerzy Bańbura

**Affiliations:** 1 Department of Experimental Zoology and Evolutionary Biology, Faculty of Biology and Environmental Protection, University of Łódź, Banacha 12/16, 90–237 Łódź, Poland; 2 Department of Biodiversity Studies and Bioeducation, Faculty of Biology and Environmental Protection, University of Łódź, Banacha 1/3, 90–237 Łódź, Poland; 3 Museum of Natural History, Faculty of Biology and Environmental Protection, University of Łódź, Kilińskiego 101, 90-011 Łódź, Poland; 4 Department of Ecology and Vertebrate Zoology, Faculty of Biology and Environmental Protection, University of Łódź, Banacha 12/16, 90-237 Łódź, Poland

**Keywords:** Ecology, Genetics, Zoology

## Abstract

Landscape conversions induced by human activities can affect dispersal patterns of various bird species and, as a result, affect genetic structure of their populations. Genetic differentiation of bird populations may be enhanced by habitat variation, especially in urban-non-urban systems. The majority of population genetic studies focus on single species, which inflicts limitations for direct comparisons of genetic responses of avian populations to urbanization. Here, we used a set of microsatellite markers to examine genetic diversity, gene flow and population structure in two common parid species, great tits *Parus major* and blue tits *Cyanistes caeruleus* occupying three sites in habitats with contrasting urbanization level in central Poland. We found low but significant divergence of urban park population with both suburban and non-urban forest great tit populations, while no differentiation was found between suburban forest and non-urban forest populations. In contrast, no evidence for genetic differentiation was found between blue tit populations from the urban park, suburban forest and non-urban forest sites. We conclude that great tits and blue tits respond to urbanization-related changes in a different way, which may be a result of different rates of migration and/or dispersal, likely higher in blue tits. Some impact may be also induced by interspecific competition. We suggest that changing the focus of urban genetic research from single to multiple species may provide novel insights into how natural populations respond to the processes of urbanization.

## Introduction

The natural environment has been extensively modified by humans^1,2^. Particularly, urbanization processes constitute relatively rapid and long-lasting alterations in the environment, triggering a major threat to wildlife^3–5^. Primarily, modern management of urban areas exerts a great impact on habitats, consequently leading to their loss, rearrangement and/or fragmentation^6,7^. Moreover, the great majority of urbanized areas have strongly altered energy flux, nutrient cycles, hydrology and temperature balance as well as increased levels of chemical, noise and light pollutions^2,8,9^. It is thought, that such habitat conversions affect population genetic diversity and structure of many terrestrial species. Even for highly-mobile organisms such as birds, which can easily avoid isolated and unfavorable habitats and fly over “city barriers”, this kind of genetic effects can also be found^5,10–17^. The greater landscape modification occurs, including mainly roads, housing, commercial property development and other anthropogenic pressures, the greater are limitations in movements of organisms and interactions between them, which ultimately can contribute to genetic isolation^9,18,19^. Hence, this factor can usually strongly impede gene flow and change allele frequencies, mainly leading to genetic diversity loss within isolated populations and increase of genetic differentiation between isolated populations^12,16,20,21^.

There is an increasing body of research on how landscape conversions made by humans affect population genetics of different species. So far, evidence for urbanization-driven genetic divergence across metapopulations was found for various bird species from different phylogenetic lineages^2,4,12,13,22^. On the other hand, little or no genetic divergence of wild bird populations in human-altered habitats has also been reported^1,19,23–26^. Thus, it seems that the patterns of genetic diversity and genetic structure across urban and non-urban bird populations are heterogeneous and possibly species- or location-specific^27^, although urbanization is often evoked as a crucial trigger in the processes of diversification. To get more comprehensive understanding of these mechanisms, factors such as life history of the species and its dispersal capability, as well as the size, spatial layout and age of habitat patches, and intensity of environmental conversions made by humans should be taken into consideration in further genetic studies on avian populations^2,9,12,13,19^.

In this study we aimed to investigate population genetic patterns in two small passerine species, great tit *Parus major* and blue tit *Cyanistes caeruleus*. Both are widespread in the Western Palearctic and, although evolved as woodland species, they nowadays abundantly inhabit various types of environments, from natural deciduous and/or mixed forests to heavily urbanized cities^28–30^. Having high dispersal potential and ability to colonize and adapt to new habitats, both the great tit and blue tit are considered as model organisms used to examine adaptation to environmental heterogeneity^26,31,32^. However, genetic studies on dispersion, migration and connectivity between populations of tits from various habitats and across a gradient of urbanization (urban and forest) are scarce and apparently inconsistent^30^. While a recent large-scale study reported that great tits form a single patchy metapopulation across Europe^32^, some other presented subtle genetic differentiation among examined populations at a fine-scale^2,5^. The results for the blue tit seem to be more consistent and usually provide support for a weak genetic differentiation among neighboring populations of blue tits in deciduous and evergreen habitats^33–35^. Surprisingly, overwhelming majority of population genetic studies of birds are conducted on a single species^12^, which does not allow for direct comparisons of genetic processes at the inter-specific level. Thus, here we applied a set of microsatellite markers to examine and compare genetic diversity and population genetic structure of great tits and blue tits occupying study sites located in habitats of varying urbanization level in central Poland**.** We considered isolation by distance as our primary hypothesis, where geographically closer populations of both species would be genetically more similar compared to those located farther apart. Alternatively, we hypothesized that genetic differentiation and population genetic structure may be produced by isolation due to landscape (urbanization) variation, where the strongest differentiation would be expected between urban and non-urban populations irrespectively of geographical distances between them, while suburban populations are expected to intermediately differentiate from the others.

## Results

All ten microsatellite loci analyzed showed a moderate to high level of polymorphism in both tit species, with the number of alleles ranging from 8 to 18 for great tits and from 5 to 32 for blue tits. The observed heterozygosity values were similar for great tits and blue tits and ranged from 0.60 to 0.94 and from 0.52 to 0.92, respectively (Table1). We found no evidence for linkage disequilibrium between any pair of analyzed loci. None of the loci departed from HWE in any of the populations. For both species, frequency of null alleles at each locus was low ≤ 0.055 (Table 1) and we found no evidence of scoring errors due to stutter bands or large allele dropout in any locus. Consequently, we retained all loci in further analyses.

**Table 1:** Summary of ten microsatellite loci characteristics for the great and blue tits including number of alleles per locus (N_a_), number of individuals (N), allele size ranges, expected heterozygosity (H_e_), observed heterozygosity (H_o_) and frequency of null alleles (NullF).

Species	Locus	N_a_	N	Size range (bp)	H_e_	H_o_	NullF
Great tits *Parus major*	Pca8	18	85	186–222	0.91	0.94	− 0.024
	PmaC25	10	85	304–334	0.87	0.93	− 0.037
	PmaD22	16	85	388–448	0.90	0.82	0.038
	PmaGAn27	17	85	201–264	0.91	0.89	.004
	PmaGAn30	8	85	294–308	0.67	0.69	− 0.039
	PmaGAn40	8	85	406–424	0.57	0.60	− 0.021
	PmaTAGAn71	11	85	170–210	0.81	0.81	− 0.008
	PmaTAGAn86	14	85	140–192	0.85	0.81	0.026
	PmaTGAn42	11	85	246–294	0.83	0.81	0.008
	PmaTGAn45	11	85	277–313	0.67	0.61	0.029
Blue tits *Cyanistes caeruleus*	Pca8	32	48	260–364	0.96	0.90	0.029
	PmaC25	8	48	316–343	0.73	0.79	− 0.055
	PmaD22	22	48	432–478	0.94	0.88	0.031
	PmaGAn27	12	48	198–258	0.88	0.92	− 0.023
	PmaGAn30	5	48	290–300	0.55	0.52	0.008
	PmaGAn40	8	48	406–430	0.66	0.60	0.049
	PmaTAGAn71	20	48	198–302	0.89	0.87	− 0.009
	PmaTAGAn86	8	48	114–128	0.79	0.71	0.054
	PmaTGAn42	9	48	254–290	0.88	0.88	− 0.001
	PmaTGAn45	16	48	303–357	0.90	0.85	0.023

The assessment of differences in genetic diversity between populations showed that they were largely similar to each other, both for great tits (Table 2) and blue tits (Table 3). Allelic richness and number of effective alleles were quite homogenous between populations within each species, with slightly, but significantly higher values recorded for blue tit (allelic richness: P = 0.03). Mean observed heterozygosity was similar across populations (Tables 2 and 3) and showed no significant variation between species (P = 1.00), similarly to the inbreeding coefficient (P = 0.13). All populations of blue tits were characterized by a higher number of private alleles (Table 3) in comparison to populations of great tits (Table 2).

**Table 2:** Summarized statistics of genetic descriptors for great tits populations including number of individuals (N) per population, number of alleles (N_a_), number of effective alleles (N_e_), heterozygosity indices (H_o_ and H_e_), inbreeding coefficient (F_IS_) and number of private alleles (P_a_).

Population	N	N_a_	N_e_	H_o_	H_e_	F_IS_	P_a_
Urban (Łódź parkland areas)	33	9.40 (0.92)	5.45 (0.73)	0.76 (0.05)	0.77 (0.04)	0.012 (0.026)	13
Suburban (Łagiewniki Forest—Łódź)	34	9.90 (0.95)	5.91 (0.83)	0.81 (0.04)	0.79 (0.04)	− 0.028 (0.031)	13
Non-urban (Spała Forest)	18	8.40 (0.88)	5.61 (0.81)	0.82 (0.04)	0.78 (0.03)	− 0.051 (0.021)	8

Mean values with standard errors provided in parentheses were calculated for 10 microsatellite loci. Urban, suburban and non-urban populations are represented by individuals sampled in Łódź parkland areas, Łagiewniki Forest, and Spała Forest, respectively.

**Table 3:** Summarized statistic of genetic descriptors for blue tits populations including number of individuals (N) per population, number of alleles (N_a_), number of effective alleles (N_e_), heterozygosity indices (H_o_ and H_e_), inbreeding coefficient (F_IS_) and number of private alleles (P_a_).

Population	N	N_a_	N_e_	H_o_	H_e_	F_IS_	P_a_
Urban (Łódź parkland areas)	16	9.50 (1.38)	6.23 (0.98)	0.78 (0.04)	0.79 (0.04)	0.020 (0.043)	15
Suburban (Łagiewniki Forest—Łódź)	20	10.80 (1.70)	6.72 (1.30)	0.81 (0.05)	0.79 (0.04)	− 0.027 (0.025)	20
Non-urban (Spała Forest)	12	9.00 (1.40)	6.85 (1.28)	0.79 (0.07)	0.79 (0.04)	0.016 (0.057)	10

Mean values with standard errors provided in parentheses were calculated for 10 microsatellite loci. Urban, suburban and non-urban populations are represented by individuals sampled in Łódź parkland areas, Łagiewniki Forest, and Spała Forest, respectively.

**Figure 1:**
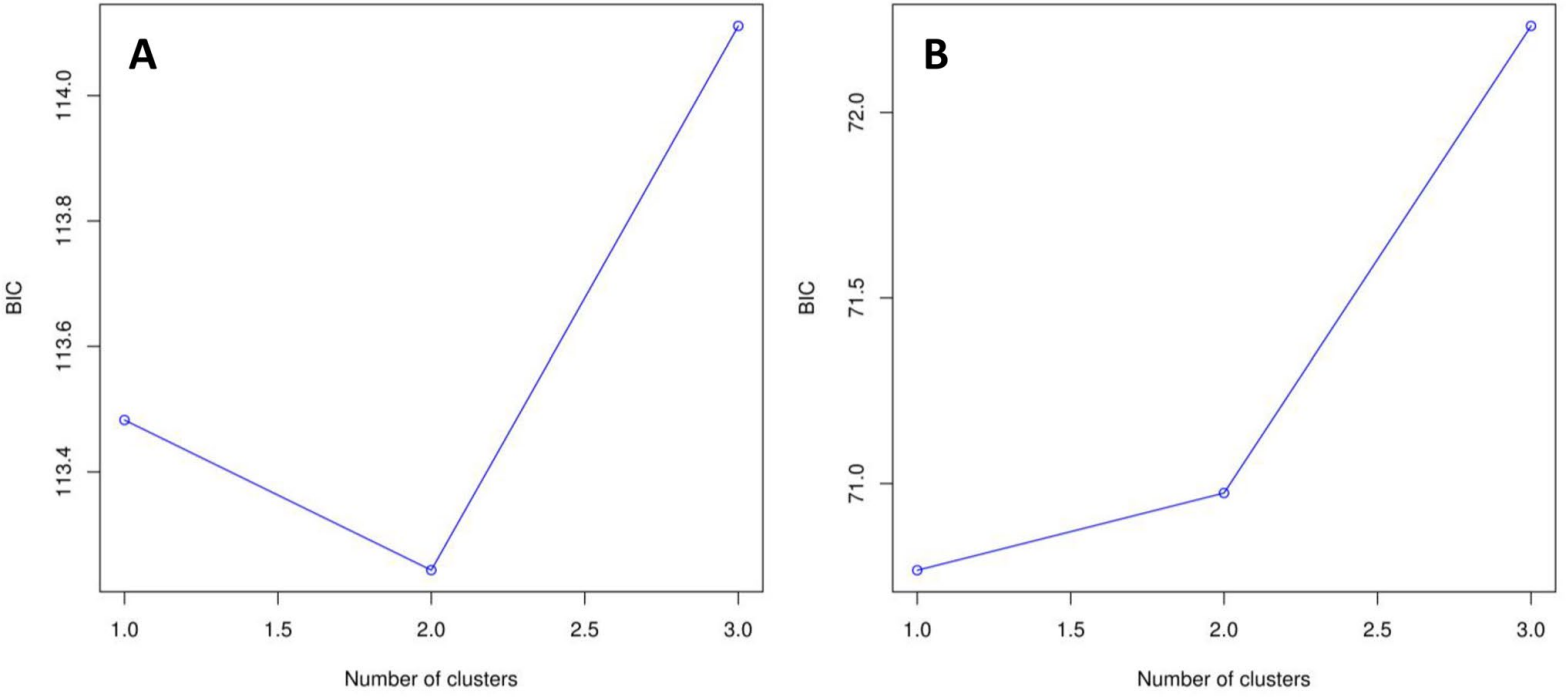
The number of genetic clusters in the great tit (**A**) and blue tit (**B**), as inferred with the lowest BIC criterion in the Discriminant Analysis of Principal Components (DAPC). Figures were created in R v.3.6.3 statistical environment (https://www.R-project.org).

While the analysis of F_ST_ provided no evidence for significant genetic differentiation between blue tit populations (all pairwise F_ST_ and F′_ST_ values non-significant, Table 4), in the great tits, we recorded a significant divergence between the urban parkland population versus the Łagiewniki Forest and the Spała Forest populations, with higher values of F_ST_ and F′_ST_ in the latter comparison (Table 5). At the same time, no evidence for significant differentiation was found between the Łagiewniki Forest and the Spała Forest populations of great tits (Table 5). A similar pattern was revealed by the analysis of pairwise Nei’s genetic distances, as comparisons of the urban parkland with the Łagiewniki Forest and Spała Forest populations of great tits were associated with greatest genetic distances (Table 6). An analysis of F_ST_ under uniform sample sizes for both species provided an evidence for significant differentiation between urban parkland versus non-urban forest populations of great tits. In this comparison, both pairwise F_ST_ and F′_ST_ values (0.01 and 0.07, respectively) and pairwise Nei’s genetic distance (Table 6) were distinctively higher compared to those for blue tits. The Discriminant Analysis of Principal Components (DAPC) supported stronger differentiation between great tit than blue populations, indicating the presence of two genetic clusters in the first species and the presence of only a single cluster in the latter (as identified by the lowest BIC criterion; Fig. 1). However, the assignment of individuals to cluster was rather inconclusive with respect to urbanization level. The Mantel test revealed that there was no significant isolation by distance in either great tits (P = 0.67) or blue tits (P = 0.35).

**Table 4:** Matrix of pairwise F_ST_ (below diagonal) and F′_ST_ (above diagonal) estimates for ten microsatellite loci between studied populations of the great tits.

Population	Urban (Łódź parkland areas)	Suburban (Łagiewniki Forest—Łódź)	Non-urban (Spała Forest)
Urban (Łódź parkland areas)	–	**0.025**	**0.047**
Suburban (Łagiewniki Forest—Łódź)	**0.005**	–	0.014
Non-urban (Spała Forest)	**0.010**	0.003	–

Bold entries highlight significant values. Urban, suburban and non-urban populations are represented by individuals sampled in Łódź parkland areas, Łagiewniki Forest, and Spała Forest, respectively.

**Table 5:** Matrix of pairwise F_ST_ (below diagonal) and F′_ST_ (above diagonal) estimates for ten microsatellite loci between studied populations of the blue tits.

Population	Urban (Łódź parkland areas)	Suburban (Łagiewniki Forest—Łódź)	Non-urban (Spała Forest)
Urban (Łódź parkland areas)	–	0.013	0.016
Suburban (Łagiewniki Forest—Łódź)	0.002	–	0.023
Non-urban (Spała Forest)	0.003	0.004	–

Urban, suburban and non-urban populations are represented by individuals sampled in Łódź parkland areas, Łagiewniki Forest, and Spała Forest, respectively.

**Table 6:** Matrix of Nei’s unbiased genetic distance for all ten microsatellite loci between selected populations of the great tits (below diagonal) and the blue tits (above diagonal).

Population	Urban (Łódź parkland areas)	Suburban (Łagiewniki Forest—Łódź)	Non-urban (Spała Forest)
Urban (Łódź parkland areas)	–	0.010	0.013
Suburban (Łagiewniki Forest—Łódź)	0.019 (0.026)	–	0.016
Non-urban (Spała Forest)	0.036 (0.054)	0.011 (0.026)	–

For great tits, values estimated under reduced sample size (uniform with blue tit) are shown in parentheses. Urban, suburban and non-urban populations are represented by individuals sampled in Łódź parkland areas, Łagiewniki Forest, and Spała Forest, respectively.

## Discussion

Our analysis of microsatellite data in two species of tits revealed that while there was no clear evidence for genetic differentiation between urban and non-urban blue tit populations, low but significant divergence was found in the great tit, as shown by F_ST_ values and Nei’s unbiased genetic distance. Specifically, great tits sampled at the urban parkland site (Łódź) were genetically diverse and distant than those from both sub- and non-urban forest sites. At the same time, both forest populations of the great tit were not genetically different from each other. The lack of genetic divergence between two distant forest (sub-urban and non-urban) populations in great and blue tits indicates that both species maintain high dispersal potential and population connectivity within study sites representing similar types of habitat. On the other hand, the gene flow between study sites located in contrasting habitats was apparently reduced in the great tit (significant divergence between urban and forest populations), when compared with the blue tit (no divergence between urban and forest populations), suggesting that the population structure of the latter species may be less responsive to the level of landscape urbanization. At the same time, for neither of the species we found evidence for isolation by distance, which suggests that, particularly in the case of great tits, genetic differentiation recorded among the study sites could not be primarily attributed to their geographical location (and distances between them), but was likely produced by variation in the landscape characteristics, e.g. the level of urbanization.

Low but significant genetic differentiation between urban and forest populations of great tits recorded in this study may seem to be inconsistent when compared to some other studies analyzing the pattern of genetic differentiation of this species in urbanization gradients. For example, Björklund et al.^5^, using microsatellite markers, found stronger significant genetic differentiation among great tits sampled at 12 parks in central Barcelona (Spain) and in an oak forest nearby the city. The average value of F_ST_ = 0.067 recorded in the Barcelona meta-population was much higher than values in our study (see Table 4), even despite the fact that distances between study sites in Spain were smaller compared to ours. We suppose that one of the reasons could be higher isolation of birds, as the study sites in Barcelona seem to be in general more separated by city infrastructure and more strongly affected by anthropogenic pressure. To better understand the discrepancies of these two studies, one should refer to a large-scale research on population genetics in the great tit by Lemoine et al.^32^. The authors compared great tit populations across Europe, showing low but significant genetic differentiation among sites (F_ST_ = 0.008). Notably, this differentiation was higher in south-western European great tits in comparison to northern parts of the continent, suggesting an effect of geographic location and important role of various environmental factors (in this particular case, autumn–winter temperatures). According to Lemoine et al.^32^, south-western populations could be more isolated and could experience stronger genetic drift and/or selective pressure effects compared to the northern populations that considered as more homogenous. In this latter case, it was supposed that gene flow determined demographic and evolutionary aspects. Specifically, habitat selection and assortative mating system may play a crucial role in the local adaptation. Therefore, it seems that conclusions presented by Lemoine et al.^32^ to some extent explain the differences found between our study (more northern population) and the study by Björklund et al.^5^ on a southern Spanish population.

Microsatellite differentiation was also analyzed in a small, structured population of great tits from Vlieland Island and a large mainland population located in the central Netherlands^36^. Again, similarly to our findings, the authors recorded low but statistically significant genetic differentiation between populations breeding in the eastern and western part of the island (F_ST_ = 0.011), despite the relatively short distance among the sites (a few kilometers away). Surprisingly, no difference was noted between the western part of Vlieland and the mainland population, while the eastern part and mainland differed significantly (F_ST_ = 0.0084). These genetic differences among both parts of the island possibly reflected an effect of unequal proportion of gene flow from mainland population. Presumably, in that case the differences in immigration rate among sites play more important role rather than differences in adaptation mechanisms^36^. In another study, conducted by Perrier et al.^2^, it was found that the urbanization induced low but significant effects on great tit genetic structure along an urban–rural gradient (average value of F_ST_ = 0.007). Once more, it was consistent with our findings, however in this particular case it was demonstrated by applying RAD sequencing techniques.

Finally, similar conclusions were also presented in the latest study by Salmón et al.^37^, where genomic responses of great tits to urbanization were examined at the broad geographical scale. An analysis of genome-wide SNP data revealed relatively low genetic differentiation between paired urban–rural populations, with values of F_ST_ ranging between 0.004 and 0.050, although this differentiation was associated with repeated polygenic responses to urban habitats that mostly included polymorphisms at genes related to neural function and development^37^.

The lack of clear evidence for any genetic differentiation among blue tit populations in our study system stands in some contrast to our findings on great tits. In our study system, extensive dispersal movements among both habitats were more likely in the blue tit, in particular from urban site, which could contribute to increased gene flow and observed homogenization of gene pools across all study populations. We suppose that interspecific competition between great and blue tits could contribute to variation in dispersal among our study sites. According to Dhondt^38^, blue tits are a weaker competitor during the breeding season than great tits, but they are also more sensitive to biotic and abiotic factors compared to great tits. Therefore, we assume that migration could be more frequent in blue tits, which has already been recorded as a partial migrant species in both central and northern Europe^39,40^, triggering more gene flow and, consequently, less pronounced genetic differentiation between populations, as we found in our study. Perhaps, it may also be important that blue tits are thought to be more sensitive to human alterations in the structure of habitats compared to great tits, at least in central Europe^30,41,42^. Another thing worth considering is that great and blue tits also compete for roosting site during wintering. Dhondt and Eyckerman^43^ found that great tits exclude blue tits from these sites, thus probably reducing their survival rates. Therefore, we suspect that such an effect also occurs in our study system. During winter periods between 2013 and 2016, we found that recoveries of banded individuals were more frequent in great tits than in the case of blue tits, with frequencies being 20% and 8%, respectively^(unpublished data)^. This suggests that more blue tits winter outside the breeding grounds, perhaps resulting in more opportunity to mix with other populations. We suppose that a weaker tendency of southern tit populations to migrate during the non-breeding season may support local population differentiation.

Other microsatellite-based studies of blue tit populations from various habitats found more genetic differentiation. Porlier et al.^33^, demonstrated a significant level of genetic differentiation among populations sampled in Corsica and in southern continental France. Pairwise estimates of F_ST_ ranged from 0.003 to 0.049 for all pairwise comparisons between these populations. According to Porlier et al.^33^, neither geographic distance nor physical barriers were the reason, but habitat type (deciduous or evergreen) and, presumably, habitat quality were identified as crucial factors. Porlier et al.^33^ concluded that their results suggest an importance of local adaptation in shaping the genetic structure of blue tits from Corsica. Low but significant genetic differentiation among blue tit populations from fragmented habitats (ca. 20 km distant) was also found in Spain, with values of F_ST_ ranging from 0.005 to 0.008^43^. According to Ortego et al.^44^, there was some evidence that habitat fragmentation may be responsible for weak dispersal movements between habitat patches, which reduced gene flow between populations.

Evidence for various effects of urbanization on population genetic structure is rapidly accumulating for an increasing number of bird taxa. Deleney et al.^12^, showed that progressing urbanization in southern California significantly reduced gene flow in the wrentit *Chanaea fasciata*, leading to a decrease in genetic diversity and significant genetic divergence among isolated groups (average value of F_ST_ = 0.095). Similar patterns were found in song sparrows *Melospiza melodia* inhabiting metropolitan Seattle, where urban development limited connectivity between sub-populations^13^. This was manifested in overall low but significant genetic divergence among study sites, with pairwise F_ST_ values ranging from 0.001 to 0.028.

Similar conclusions can be drawn from studies conducted on house sparrows *Passer domesticus*^22^ and blackbirds *Turdus merula*^4^. The first study showed genetic differences among urban and rural populations of house sparrow around Ghent (Belgium) and demonstrated that average relatedness was higher among urban birds compared to those from rural habitats^22^. In contrast, urban populations of blackbirds were characterized by the lower genetic diversity compared to paired rural populations. At the same time, differentiation among urban blackbird populations proved to be greater than that between urban and rural populations^4^. In turn, analyzes of microsatellite data conducted by Brewer et al.^19^ revealed that the level of urbanization did not affect genetic diversity and genetic structure of song sparrows inhabiting patches in a gradient of urbanization around Blacksburg (USA), where pairwise F_ST_ values ranged from 0.011 to 0.023. Similar conclusion was provided by studies on black headed gulls *Chroicocephalus ridibundus* in northern Poland^15^ and on common kestrels *Falco tinnuculus* in Czech Republic^24^, where urban and rural populations did not differ genetically.

In summary, to our best knowledge this study is the first attempt to examine the genetic diversity and population genetic structure of both great tits and blue tits co-occupying contrastingly different yet spatially close habitats. Our data indicate that there was a contrasting pattern of the genetic structure among the two parid species. This can suggest, that both great and blue tits respond differently to urbanization factors, presumably due to a different level of migration and/or dispersal capability of urban populations. We suppose that in our study system these discrepancies in movements may be in some part related to interspecific competition between great and blue tits, which could already start during winter time preceding the breeding season. It is necessary to emphasize that our conclusions were based on data from three particular study sites located in a single geographical region (central Poland). Although we think that our results can possibly reflect more general patterns of great tit and blue tit responses to urbanization, it cannot be directly inferred from our data. We are also aware that our study could have suffered from relatively low sample sizes and from relatively few genetic markers used in our analyses. Despite these methodological limitations, we consider our analyses and conclusions reliable, as our sample sizes were actually comparable (or even more extensive) to other genetic studies on tits^5^, and small panels of microsatellite markers have been reported to produce highly consistent results with genomic approaches based on thousands of SNP loci^45–47^. Nonetheless, we acknowledge that further research should incorporate more comprehensive sampling (possibly at a broader geographical scale) and more robust genotyping approaches (possibly combining genetic and genomic methodology) to overcome the limitations of the current study and provide more general insights into urbanization processes of the two most common European parid species.

## Materials and methods

### Study site, sampling and genotyping

This study was conducted as part of a long term project (initiated in 1999) on the breeding biology of hole-nesting passerines in central Poland. The study was carried out in compliance with the ARRIVE guidelines. All procedures were performed in accordance with Polish legislation and approved by the Local Bioethical Commission for Experiments on Animals, Medical University in Łódź (No. 70/ŁB07/2015) and the Regional Directorate for Environmental Protection (WPN-II.6401.13.2016.MS).

**Figure 2:**
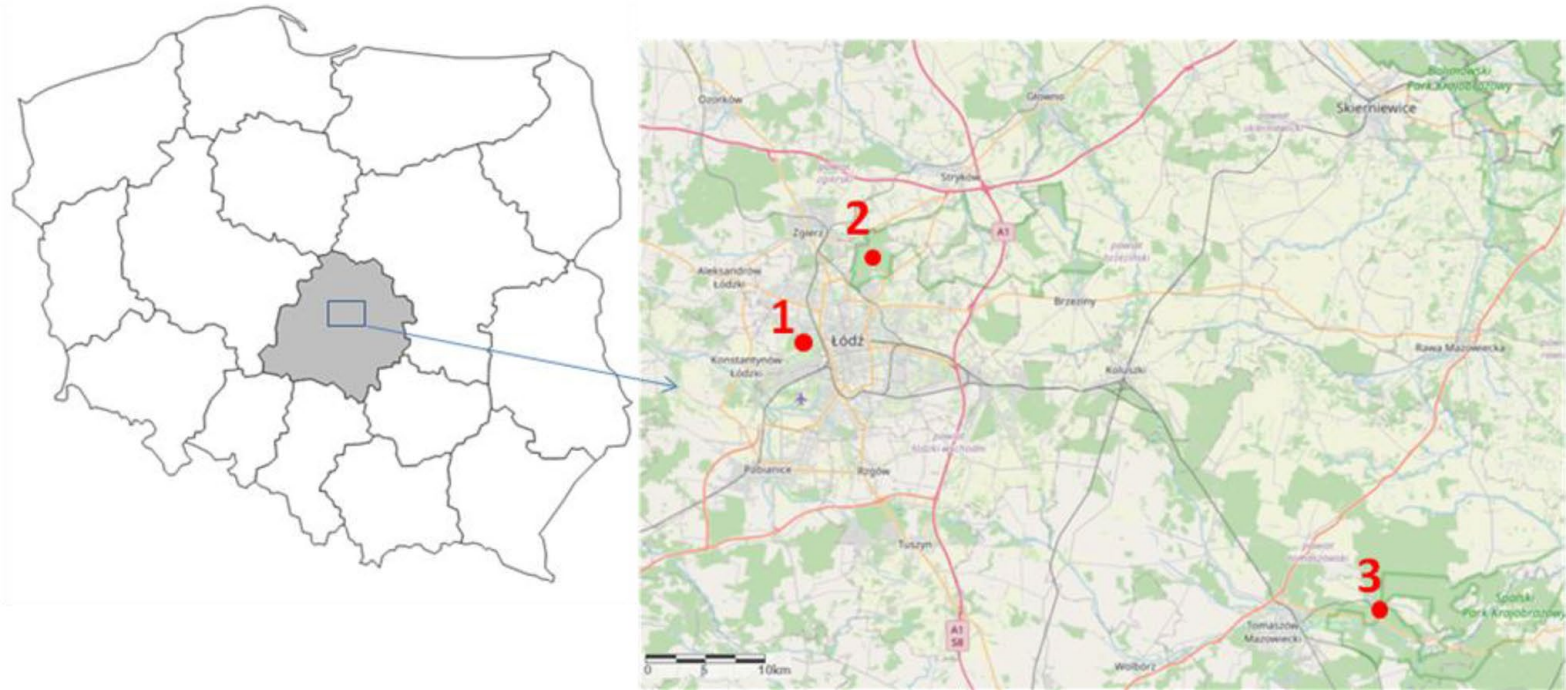
Map of the study area showing the spatial location of the study sites (1—urban parkland; 2—suburban (Łagiewniki) Forest; 3—urban (Spała) Forest). The map of Poland was generated by applying Map function ver. 3.1.85 implemented in STATISTICA software^71^ The map with marked study sites was created using QGIS 3.16.3 Hannover software (https://www.qgis.org/). Both maps were merged and edited in GIMP v. 2.10.10 (GIMP Development Team 2019: http://gimp.org).

During the 2018 breeding season, a total of 85 and 48 individuals of great and blue tit, respectively, were sampled at three study sites (Fig. 2): two were located around Łódź, in two contrasting habitats (urban parkland and mature deciduous forest) and the third one was situated in the Spała Forest (51°32′ N, 20°07′ E). The urban parkland site is located in the southwest part of Łódź (51°45′ N, 19°24′ E) and comprises botanical and zoological gardens, covering approximately 80 ha^48^. This area has highly fragmented arrangement of tree cover, formed artificially. The forest site encompasses ca. 140 ha, situated in the interior of the Łagiewniki Forest (51°50′ N, 19°29′ E), bordering to the northeast part of the suburbia of the city of Łódź. This study site consists of tree stands mainly based on mature deciduous trees, with oaks *Quercus robur* and *Quercus petraea* being dominant species^49^. The third study site was established in the Spała Forest District located within the Pilica valley, which is situated ca. 50 km apart from Łódź (Fig. 2). The Spała Forest belongs to the largest managed and natural forest areas in Łódź Province. Dominant stand-species in this area constitute *Pinus silvestris* (90%) with marginal participation of old oaks (2.3%) which can be mainly found in one of nature reserves (Spała), within the Spała Landscape Park^50^. Referring to the data collected and presented in some of the our previous studies^51,52^, we emphasize that both the study sites located in Łódź (urban parkland and suburban forest) differed distinctly not only in habitat structure but also in the level of anthropogenic pressure. This was mainly manifested by apparent differences in a variety of measures of human presence and activity in these areas, with much higher human attendance being recorded in the urban parkland site (see details in Glądalski et al.^51^). Additionally, the urban parkland site was situated relatively close to the city center (ca. 3 km) and surrounded by roads with intense traffic as well as city buildings, whereas the study site located in the Łagiewniki Forest was characterized by a lower number of roads with many traffic restrictions^52^. At the same time, the study site in the Spała Forest District is remote from dense human settlements and is surrounded by a landscape protection area. As a consequence, human presence at the site is highly limited, both in terms of their abundance and disturbance to breeding birds^50^ own.obs.

From each sampled bird, a sample of ca. 10 µl of blood was collected from the ulnar vein on FTA MiniCards and, after drying, stored at room temperature for the purpose of genetic analyses. Population genetic structure of great and blue tits was examined using microsatellite markers, which are recognized as a relevant tool, traditionally used in population genetic and evolutionary studies^53,54^. Both the great and blue tit individuals were genotyped at ten microsatellite loci (Table 1), originally developed for blue tits (Pca8; Dawson et al.^55^) and great tits (PmaC25, PmaD22, PmaGAn27, PmaGAn30, PmaGAn40, PmaTAGAn71, PmaTAGAn86, PmaTGAn42, PmaTGAn45; Saladin et al.^54^). DNA extraction was performed using GeneMATRIX Bio-Trance DNA Purification Kit. A part of every dried blood sample (approx. 2 mm^2^) was cut away from each FTA MiniCard, with the use of sterile cutter. Subsequently, DNA isolation was performed in accordance with the manufacturer’s protocol guidelines. In the next step, polymerase chain reaction was conducted using the final volume of 20 µl containing 10 µl of DreamTaq PCR MasterMix (Thermo Fisher Scientific) and 0.5 µl of primer and 1 µl of extracted DNA. The amplification steps and conditions followed the protocols developed by Dawson et al.^55^ and Saladin et al.^54^. One exception was applied to the annealing temperature for PmaTAGAn86 primers, which finally was set to 61 °C. Forward primers were labeled with 6-FAM fluorescent dye and PCR amplifications were performed separately for each locus. Microsatellite genotyping was carried out in a commercial laboratory (Genomed, Warsaw, Poland). Allele sizes were determined against an internal lane size standard (Henescan TM 600 LIZ) using Geneious software.

### Microsatellite loci—genetic variability

The Micro-Checker 2.2.3 software^56^ was used to check for any mistyped allele sizes and scoring errors due to large allele dropout or stuttering. Deviation from Hardy–Weinberg equilibrium (HWE) was tested for each locus in each population with the Markov-chain algorithm (chain length: 1,000,000; number of dememorization steps: 100,000) implemented in the exact tests^57^ available in Arlequin 3.5.1.2^58^. Linkage disequilibrium between all pairs of loci was tested in FSTAT 2.9.3.2^59^. The results of these analyses were adjusted for multiple testing with Bonferroni correction^60^. The expected and observed heterozygosity (H_o_ and H_e_, respectively), allelic richness (N_a_) and the frequency of null alleles were computed for each locus using Cervus 3.0.3 software^60^, while inbreeding coefficients (F_IS_) were estimated using GeneAlEx 6.5 software^61,62^. To examine differences in genetic diversity measures (N_a_, H_o_, and F_IS_) between species, permutation procedure (15,000 permutations) was applied by using FSTAT software.

### Genetic structure

Evaluation of genetic divergence between populations of each species was conducted by calculation of Wright’s fixation index F_ST_. This is a classic measure of population divergence, although it is known to diminish the role of mutations in inducing genetic variation within populations^15,63^. Therefore, an alternative statistic R_ST_^63^ was introduced based on the stepwise mutation model, which can more accurately reflect changes in microsatellite allele sizes^21^. However, under certain scenarios, R_ST_ can be less accurate in reflecting population divergence than F_ST_ due to high sampling variance^21^. Taking all this into consideration, to check whether microsatellite allele sizes provide information on population differentiation within our dataset, we applied the allele size permutation tests^64^, using SPAGeDi 1.5 software^65^. The tests were run under the null hypothesis (H_o_) that variation in allele sizes does not affect population differentiation (F_ST_ = R_ST_; 20,000 permutations)^64^. The results did not allow to reject the null hypothesis both for the great tit (P = 0.41) and blue tit (P = 0.82). Hence, we used F_ST_ statistic as a measure of genetic differentiation for both species and pairwise F_ST_’s were computed with 100,000 permutations in Arlequin 3.5.1.2 software. Additionally, standardized F_ST_ values (F′_ST_) were calculated as the original F_ST_ divided by the maximum possible F_ST_ value in respect to the intra-population genetic variation. These computations followed recommendations by Hedrick^66^ and were performed using RecodeData 0.1 software^67^. To measure the genetic distance between the populations we also calculated pairwise Nei’s unbiased genetic distances^68^, using GeneAlEx 6.5 software. We also checked whether any differences in the genetic structure between great and blue tits were related to inequality in sample sizes. For this purpose, we re-computed F_ST_ statistics and pairwise Nei’s unbiased genetic distances for great tits, randomly limiting their sample sizes for each population to match them with those available for blue tits. To examine the effects of geographical distance on genetic structure we conducted Mantel test between all pairwise F_ST_ and the corresponding pairwise geographical distances, with 10,000 permutations using the package *ade4*^69^ developed for R statistical environment. Finally, we conducted the Discriminant Analysis of Principal Components (DAPC) to infer the number of genetic clusters within our data and to assign individuals to these clusters. The analysis was run in the *adegenet*^70^ R package. The number of genetic clusters (1 ≤ *k* ≤ 3) were identified based on the minimum Bayesian Information Criterion (BIC) using the same number of principal components (PCs) for each species (n = 40). In the case of *k* > 1 we performed the discriminant analysis an posterior assignment of individuals to cluster using *dapc* function.

## Supplementary Information

Supplementary Information.
